# Age-Related Macular Degeneration (AMD): A View to the Future

**DOI:** 10.3390/jcm10051124

**Published:** 2021-03-08

**Authors:** Joan W. Miller, Lily L. D’Anieri, Deeba Husain, John B. Miller, Demetrios G. Vavvas

**Affiliations:** Retina Service, Department of Ophthalmology, Harvard Medical School, Massachusetts Eye and Ear, Boston, MA 02114, USA; lily_danieri@meei.harvard.edu (L.L.D.); Deeba_Husain@meei.harvard.edu (D.H.); john_miller@meei.harvard.edu (J.B.M.); Demetrios_Vavvas@meei.harvard.edu (D.G.V.)

Age-related macular degeneration (AMD) is the leading cause of blindness in people over age 50 worldwide, and the third leading cause of blindness overall. As the world population ages, AMD is expected to grow in prevalence, with the number of estimated cases predicted to reach 288 million by 2040 [1]. AMD is typically classified into two forms—a non-neovascular, or “dry” form and a neovascular “wet” form, but even these simple terms are evolving as we incorporate new information from optical coherence tomography-angiography (OCT-A).

All AMD starts as the dry form and is characterized by accumulation of deposits under the retinal pigment epithelium (RPE) and neurosensory retina, as well as degeneration of the RPE, photoreceptors, and even the choroidal vasculature, whereas wet AMD is characterized by the development of new choroidal vessels that are often highly permeable and fragile. This choroidal neovascularization (CNV) extends from the choroid into the subretinal space within Bruch’s membrane, or in the subretinal RPE space [2].

Severe vision loss and impairment of activities of daily living most often occurs in advanced AMD due to two primary causes: 1. central geographic atrophy (GA), in which large sections of RPE and overlying photoreceptor cells are lost; or 2. CNV with its associated leakage of subretinal fluid, lipid deposition, hemorrhage, RPE detachment, and/or fibrotic scarring [3]. AMD is a complex, multifactorial disease and the associations with genetic and environmental factors are well-recognized. While prior research has pointed to and implicated roles for numerous pathways in the pathobiology of AMD, including inflammation, complement, lipids, and cell death, our understanding of their precise mechanisms, as well as the relative contributions of these pathways, remains incomplete.

The demonstration of the critical role vascular endothelial growth factor (VEGF) plays in ocular neovascularization formed the basis for the development of successful anti-VEGF therapies for the treatment of neovascular AMD and other retinal diseases. We now have four anti-VEGF agents—brolucizumab (Beovu^®^), aflibercept (Eylea^®^), ranibizumab (Lucentis^®^), and pegaptanib (Macugen^®^)—approved by governmental agencies including the United States Food and Drug Administration (FDA), with additional agents in development. Bevacizumab (Avastin^®^) is approved by the FDA for the treatment of colorectal and other cancers, and continues to be used off-label as a therapy for nvAMD and other conditions. The use of these anti-VEGF therapies has revolutionized care for patients with neovascular AMD, and other neovascular ocular conditions such as RVO, DME, and ROP, and has dramatically improved vision outcomes and quality of life for millions of people worldwide. However, anti-VEGF therapies are only supportive treatment for AMD, and several issues remain. To date, the mechanisms underlying the pathologic processes of AMD remain elusive, and currently there are no treatments for either early or intermediate AMD, nor for the later advanced, atrophic stage of AMD. While studies have indicated multiple biological pathways in the formation and progression of dry AMD, including lipid metabolism and transport regulation, inflammation, extracellular matrix remodeling, cellular adhesion, cellular toxicity, cell death, and angiogenesis, there is not yet a unifying hypothesis that adequately explains initiation of the disease process or the subsequent progression to RPE and photoreceptor degradation. It is possible that there are different subtypes in early and intermediate AMD that are dominated by these different pathways, including lipids, autophagy, inflammation, or a combination of these pathways. Ultimately, the clinical course converges on an advanced enough form where neovascularization or atrophy occurs, and these late events may be the same across all the subtypes.

Despite experiencing improvement in vision outcomes with anti-VEGF treatment, patients with neovascular AMD ultimately lose vision, likely due to underlying continual retinal degeneration and progression of geographic atrophy. Neuroprotective therapies hold promise to slow or prevent photoreceptor cell death as it occurs. Studies have found that in-tandem inhibition of necrosis (through the RIPK pathway) and apoptosis (through the caspase pathway) can prevent photoreceptor loss [4]. Since cell death pathways are redundant and complementary, combination therapies to block both necrosis and apoptosis may be effective in dry AMD. As the Fas signaling pathway is upstream of both cell death and inflammatory signaling pathways, inhibition of Fas signaling at the receptor may represent a potential therapeutic target for the prevention of photoreceptor loss [5,6]. Recently, clinical trials were launched investigating the therapeutic benefits of blocking Fas signaling activation in patients with retinal disease, with the first patient treated in October of 2019 to determine safety and tolerability of an investigational drug in patients with a macula-off rhegmatogenous retinal detachment.

In cases where photoreceptor preservation is no longer possible, another approach is being pursued to attempt to replace those cells in order to restore and maintain vision. Work from several groups has paved the way for the use of stem cells—including human embryonic stem cells (hESCs) and induced pluripotent stem cells (iPSCs)—to treat retinal diseases, including AMD [7,8,9]. In 2019, a four-year follow-up on a neovascular AMD patient who had received an iPSC-derived retinal pigment epithelium sheet transplant in 2014 demonstrated graft survival and expansion, with no serious adverse effects related to the stem cells themselves. Additionally, the patient had stable visual acuity despite receiving no anti-VEGF treatment since transplant in 2014 [10]. Recent innovations in this area are harnessing advances in stem cell research and tissue engineering to investigate the use of retina organoids as a source of tissue for transplantation [11,12]. These studies may provide the practical tools for the optimization of transplantation strategies for future clinical applications.

Treatment of early and intermediate stage AMD is limited to lifestyle changes and nutritional supplements [13,14]. While the mechanisms underlying the pathologic processes of AMD are not fully understood, perhaps identification of therapeutic targets for these stages of AMD are complicated by multiple, not yet well-defined subtypes that are driven by different biological pathways. In identifying these subtypes, we may discover that different pathways—lipids, autophagy, or inflammation, for example—may dominate in separate subtypes in the earlier stages of AMD until they reach a cusp to converge and then progress to advanced AMD where neovascularization or atrophy occurs, with these late events occurring independent of the earlier stage subtype. Improving disease classification for early and intermediate stage AMD may aid in the identification of druggable targets, and ultimately, the development of successful therapeutic strategies. Work in this area is underway, with studies showing correlations between histopathological markers identified in postmortem eyes with clinical features observed with optical coherence tomography (OCT) [15,16]. While this is still a developing area, we can envision using a combination of validated biomarkers (imaging and systemic—including metabolomics and microbiome), clinical information, genomics, and functional testing to help elucidate and characterize different AMD subtypes, describe the natural disease history, and identify novel potential therapeutic targets.

In this Special Issue, we hope to build upon these ideas and highlight recent advances in the management and diagnosis of AMD. Our authors discuss the future of diagnosis as they examine the development and characterization of imaging and systemic biomarkers, the utilization of new imaging modalities, and improvements in functional testing. We also look at management and treatment of AMD, discussing the role of artificial intelligence (AI) and telemedicine within the clinic, and the successes and failures of potential therapeutic targets such as complement and stem cells. In addition, we discuss the molecular pathobiology of AMD, including lipids and tumor necrosis factor (TNF) and other inflammatory cytokines, as well as the role genetics plays in disease development and progression. We examine anti-VEGF therapy in more depth, including presumed “non-responders” and also inflammatory complications, and discuss the impact these issues have on clinical management and treatment of AMD.

The ultimate goal of this collection is to create a comprehensive overview of the current status of collective AMD knowledge, highlighting advances that inform management and diagnosis of AMD. This issue contains contributions from leading experts in the field, demonstrating their efforts to improve our understanding, diagnosis, and treatment of AMD, and outlining their vision for future directions. Moving forward, we hope this collection can serve as a valuable resource to guide AMD diagnosis and management.
